# Greater Protein Intake Emphasizing Lean Beef Does Not Affect Resistance Training-Induced Adaptations in Skeletal Muscle and Tendon of Older Women: A Randomized Controlled Feeding Trial

**DOI:** 10.1016/j.tjnut.2024.04.001

**Published:** 2024-04-09

**Authors:** Chad C Carroll, Nathan WC Campbell, Rebecca L Lewis, Sarah E Preston, Chloe M Garrett, Hannah M Winstone, Anna C Barker, Johnny M Vanos, Lucas S Stouder, Camila Reyes, Matthew A Fortino, Craig J Goergen, Zachary J Hass, Wayne W Campbell

**Affiliations:** 1 Department of Health and Kinesiology, Purdue University, West Lafayette, IN, United States; 2 Weldon School of Biomedical Engineering, Purdue University, West Lafayette, IN, United States; 3 School of Nursing, Purdue University, West Lafayette, IN, United States; 4 School of Industrial Engineering, Purdue University, West Lafayette, IN, United States; 5 Regenstrief Center for Healthcare Engineering, Purdue University, West Lafayette, IN, United States; 6 Department of Nutrition Science, Purdue University, West Lafayette, IN, United States

**Keywords:** MRI, tendon cross-sectional area, tendon modulus, ultrasound, clinical trial, dietary protein intake

## Abstract

**Background:**

Although experimental research supports that resistance training (RT), especially with greater dietary protein intake, improves muscle mass and strength in older adults, comparable research on tendons is needed.

**Objectives:**

We assessed the effects of a protein-rich diet emphasizing lean beef, compared with 2 control diets, on RT-induced changes in skeletal muscle and tendon size and strength in older women.

**Methods:**

We randomly assigned women [age: 66 ± 1 y, body mass index (BMI): 28 ± 1] to groups that consumed *1*) 0.8 g total protein/kg body weight/day from mixed food sources (normal protein control, *n* = 16); *2*) 1.4 g/kg/d protein from mixed food sources (high protein control, *n* = 17); or *3*) 1.4 g/kg/d protein emphasizing unprocessed lean beef (high protein experimental group, *n* = 16). Participants were provided with all foods and performed RT 3 times/wk, 70% of 1-repetition maximum for 12 wk. We measured quadriceps muscle volume via magnetic resonance imaging (MRI). We estimated patellar tendon biomechanical properties and cross-sectional area (CSA) using ultrasound and MRI.

**Results:**

Dietary intake did not influence RT-induced increases in quadriceps strength (*P* < 0.0001) or muscle volume (*P* < 0.05). We noted a trend for an RT effect on mean tendon CSA (*P* = 0.07), with no differences among diets (*P* > 0.05). Proximal tendon CSA increased with RT (*P* < 0.05) with no difference between dietary groups (*P* > 0.05). Among all participants, midtendon CSA increased with RT (*P* ≤ 0.05). We found a decrease in distal CSA in the 0.8 g group (*P* < 0.05) but no change in the 1.4 g group (*P* > 0.05). Patellar tendon MRI signal or biomechanical properties were unchanged.

**Conclusions:**

Our findings indicated that greater daily protein intake, emphasizing beef, did not influence RT-induced changes in quadriceps muscle strength or muscle volume of older women. Although we noted trends in tendon CSA, we did not find a statistically significant impact of greater daily protein intake from beef on tendon outcomes.

This trial was registered at clinicaltrials.gov as NCT04347447.

## Introduction

Sarcopenia, i.e., loss of muscle mass and strength, is a well-established consequence of normal aging. Skeletal muscle strength and mobility also depend on tendon connective tissue composition and mechanical properties. Tendons are strong fibrous connective tissues that attach skeletal muscle to bone, allowing for the effective transfer of tension developed during cross-bridge cycling. Importantly, skeletal muscle strength and function are related to tendon connective tissue biomechanical properties [1]. Specifically, greater tendon connective tissue cross-sectional area (CSA) and stiffness optimize force transfer through tendons. Optimal tendon stiffness maximizes musculoskeletal function, including muscle power output [1], locomotion economy [[2], [3], [4]], and balance control [[5], [6], [7], [8]]. Further, tendon pain and injury are among the most common musculoskeletal disorders and a significant healthcare problem, especially for older adults. Painful tendon pathologies reduce the quality of life of afflicted individuals by limiting their ability to complete activities of daily living, participate in the workforce, and engage in recreational activities [9].

As with skeletal muscle, aging leads to the decline of tendon connective tissue quality, including reductions in tendon collagen (the primary structural component of a tendon) and a decrease in tendon CSA [10,11]. Research indicates that resistance training (RT) can improve muscle mass and strength in older adults but may not counter the effects of aging on tendons [12,13]. Specifically, a 12-wk RT protocol increased tendon stiffness in young [14] but not older adults [12]. To our knowledge, no approaches are available to minimize loss of muscle mass and quality while improving tendon tissue quality and function in older adults. Therefore, a need exists to scientifically assess interventions that concomitantly improve muscle and tendon tissue strength and function. This need is highly relevant for women due to their higher risk of sarcopenia than men [15] and the reduced adaptability of their tendon tissues to exercise [16].

RT, especially when combined with higher protein intake, has consistently been shown to improve muscle mass and strength in older adults [[17], [18], [19]]. However, complementary research on tendons is lacking. Further, limited research exists on the effect of beef consumption combined with RT on sarcopenia-related outcomes, especially in older women [20]. We have recently demonstrated that oral amino acid consumption enhances peritendinous amino acids [21], indicating that nutritional interventions likely increase the bioavailability of amino acids in the local tendon environment, as reported in skeletal muscle [22]. Emerging research suggests that diets rich in leucine or glycine, as in beef [23,24], augment resistance or aerobic training-induced improvements in tendon CSA in rodents [25] and younger adult humans [26]. However, the potential benefit of dietary proteins from lean meat, such as beef, on connective tissue adaptations in older adults requires investigation. We assessed the effects of a healthy, protein-rich diet emphasizing lean beef on RT-induced changes in skeletal muscle and tendon connective tissue size, strength, and quality in older women. We hypothesized that a healthy diet high in protein emphasizing lean beef would augment improvements in skeletal muscle and tendon responses to RT compared to a healthy diet with normal or high protein and less total red meat. Our primary outcomes were quadriceps skeletal muscle volume and strength and patellar tendon CSA, MRI signal, and biomechanical stiffness and modulus.

## Methods

### Participants

**FIGURE 1:**
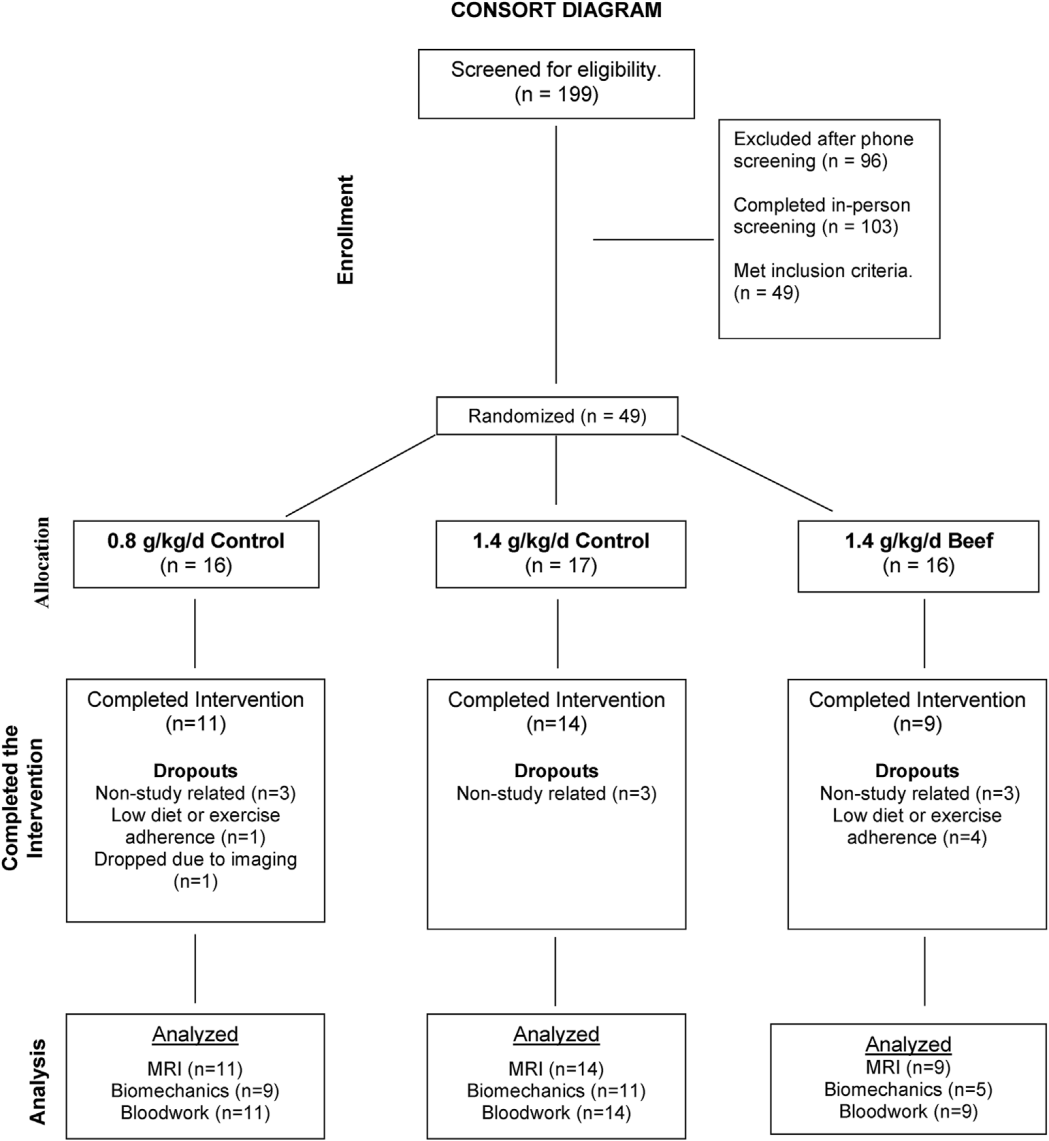
Consort diagram.

We conducted a randomized controlled trial with older adult women. Women were randomly assigned (Figure 1) to consume *1*) the United States Academy of Medicine recommended protein intake (0.8 g protein/kg body weight/day) with total protein intake coming from a variety of animal- and plant-based sources (0.8-mixed, normal protein control), a protein-rich diet (1.4 g protein/kg/d) with the additional protein content obtained from a mixture of nonred meat animal sources and plant sources (1.4-mixed, high protein control), or a protein-rich diet (1.4 g protein/kg/d) with the additional protein content obtained from unprocessed lean beef (1.4-beef, high protein experimental). We registered our project at clinicaltrials.gov (NCT04347447). The Purdue University Institutional Review Board approved this project (IRB-2019-218). We provided participants with monetary compensation for their efforts.

Before enrollment, participants completed a basic medical history. A blood sample was collected for a basic metabolic panel and lipid-lipoprotein profile. Inclusion criteria included being sedentary (≤1 d/wk of aerobic or resistance exercises for ≥1 y), having a 25–35 BMI (in kg/m^2^), and being nondiabetic. Individuals chronically consuming medications that affect protein or collagen metabolism (e.g., acetaminophen, ibuprofen, or prescription cyclooxygenase inhibitors) were excluded [12]. Individuals unable to complete the RT protocol for medical reasons (e.g., knee replacement) were also excluded. An outline of participant screening, randomization, and participation is provided in the Consort Diagram (Figure 1).

### RT protocol

All participants completed a supervised 12-wk RT program targeting the quadriceps muscles. The specific protocol chosen has been shown to increase muscle mass in young and older adults and improve tendon strength and CSA in young adults [13,14]. Specifically, participants completed 36 sessions (3 sessions/wk on nonconsecutive days) of knee extension and leg press exercises consisting of 4 sets of 10 repetitions at 70% of 1-repetition maximum (1-RM) (Technogym USA Corp.). Sets were separated by 90 s of rest. The 4 sets at 70% 1-RM were preceded by 2 “warm-up” sets of 10 repetitions at 40% of 1-RM. Per previous investigations, our lowest accepted training frequency was an mean of 2.5 sessions per week [14]. We also integrated upper-body and hamstring resistance exercises to promote whole-body physical function. Participants completed 4 sets each of leg curl, seated bench press, and seated row exercises. All training was completed in the Purdue University A.H. Ismail Center for Prevention and Lifestyle Medicine and supervised by a research team member. We asked that all participants not perform additional resistance or high-intensity aerobic training during the study period.

### Dietary intervention

During baseline, each participant’s self-chosen food and nutrient intakes were estimated on 3 nonconsecutive days (2 weekdays and 1 weekend day) using the Automated Self-Administered 24-h (2019–2021 versions) dietary assessment tool (https://epi.grants.cancer.gov/asa24). Menus were developed using Pronutra software (Viocare, Inc.). Participants were instructed to complete a daily menu checklist to track self-reported deviations from the provided foods/beverages. The group-specific dietary patterns included 7 daily menus that the participants repeatedly consumed during the 12-wk intervention period (i.e., a 7-d cycle of menus). Diet-related activities and assessments were completed with help from the NIH-supported Indiana Clinical Research Center Bionutrition facility at Purdue University (https://www.purdue.edu/hhs/nutr/crc/).

All participants consumed their usual, unrestricted self-chosen diets during screening and baseline assessments. During intervention weeks 1–12, each participant consumed prescribed diets that conformed to the United States Healthy Dietary Pattern consistent with the 2015–2020 Dietary Guidelines for Americans [27]. One control diet contained the recommended dietary allowance for total protein intake of 0.8 g/kg/d, with the protein provided from a variety of animal and plant-based sources, including lean beef (one 3-oz portion/wk), chicken, eggs, dairy, beans, grains, nuts, and seeds. The second control diet provided 1.4 g protein/kg/d from various animal and plant-based sources (excluding additional red meats). The experimental diet contained 1.4 g protein/kg/d, with the additional 0.6 g/kg/d protein predominantly from lean beef (one 3-oz portion/d; total beef intake 24 oz/wk). The energy content of the additional protein foods was isoenergetically offset by substitution for low-protein foods.

The quantities of protein consumed were chosen based on usual protein intakes among older adults in the United States [28]. Specifically, participants on the normal protein control diet consumed 0.8 g protein/kg/d, representing the 50th percentile of the range of protein intake of older adults and the current recommended dietary allowance for protein in the United States [14]. The high protein intake groups of 1.4 g protein/kg/d represent the 95th percentile of protein intake for older adults in the United States [14].

The investigative team purchased all food and beverages (except water) throughout the 12-wk intervention period. Specifically, grocery items were ordered online from a local grocery vendor. Participants picked up their orders from the vendor weekly. The research team also provided counseling on proper food portioning, preparation, and intake documentation. We incorporated various cuts of lean, unprocessed red meat into the intervention dietary pattern, as approved by the American Heart Association’s Food Certification Program. These cuts of meat include >90% lean ground beef and choice or select beef round, sirloin, chuck, and tenderloin. Lean meat contains <10 g total fat, <5 g saturated fat, and <95 mg cholesterol and is not preserved by smoking, curing, salting, or adding chemical preservatives, per the USDA definitions.

### Patellar tendon biomechanical properties

We assessed the patellar tendon tangent modulus by plotting the relationship between in vivo stress and strain [11,12,29]. To determine patellar tendon stress (σ_xx_) and strain (ε_xx_), participants were secured with the knee at 90° of flexion [11,30]. A ridged cuff was fixed around the ankle. The ankle strap was connected to a strain-gauge load cell, which interfaced with a computer to collect force data points. Participants gradually applied isometric force over 10 s to the maximum effort to minimize differences in strain rate [11,29,30]. Visual feedback of force output was provided by graphically displaying force output on a computer screen. After an initial familiarization session to establish a participant’s maximal effort and ensure that participants could increase joint moment linearly during the 10-s ramp [1,11,30], participants completed 2 additional sessions, as done previously [11,12,30]. We instructed participants to refrain from exercise for 48 h before and between tests to minimize the effect of loading history on our assessments [11,12,30].

An ultrasound transducer was affixed to the skin over the patellar tendon (L761V linear array transducer, 5-11 MHz; Sonoscope Medical Corp) to record tendon movement (AV.io HD; Epiphan Video) simultaneously during the 10-s contraction [29]. Video recordings were synchronized with the force recordings using a custom LabVIEW program version 17.0.l.f3 (National Instruments Corp.). After testing, ultrasound videos were loaded into a custom MATLAB graphical user interface. Next, manually defined points surrounding the patellar tendon were tracked across frames using speckle-tracking technology [31]. Regional tendon deformation between adjacent frames was derived from the resultant boundary-point displacement information. The 2D Green-Lagrange strain tensors were subsequently calculated relative to the initial video frame. Finally, we extracted the longitudinal (horizontal-e_xx_) strain component, the primary plane of strain from attachment to insertion, which was used to construct stress-strain plots.

We calculated a single agonist force vector in line with the patellar tendon from the load cell data by dividing the total knee extension moment at 90° (recorded force/knee external moment arm) by the internal moment arm, estimated from knee breadth [32]. The tendon stress was then calculated by dividing the force by MRI-derived CSA (force/CSA) [11,12,30]. Normal strain was plotted against stress and fitted with a polynomial function [11,12]. The tangent modulus was estimated from the final 20% of the stress-strain curve (slope of the line) [29,33]. As noted in our consort diagram (Figure 1), we could not assess tendon biomechanical properties in all participants. The analysis could not be completed due to poor ultrasound image quality, the inability of a participant to perform the ramped contraction properly, or the inability of the individual’s knee to fit properly in the knee coil [12].

### Skeletal muscle MRI

Pre- and postintervention scans were completed at the Purdue University Life Sciences MRI facility. Before scanning, participants rested in a supine position for 30 min. Images were obtained using a Siemens Magnetom Prisma 3T whole-body MRI system. Thigh images were obtained, as previously described [13]. Fifty 8-mm slices (no gap) were obtained beginning at the tibial notch and continuing proximal (Repetition Time (TR): 2000 ms, Echo Time (TE): 8 ms, field of view: 480 × 480, resolution: 512 × 512, voxel size: 0.94 × 0.94 × 8, flip angle: 120°) [13]. Quadriceps muscle volume was obtained by manually circumscribing every third slice to determine the CSA. Volume was then acquired by accounting for slice thickness. All muscle volume analyses were completed by the same investigator who was blind to the group assignments.

### Patellar tendon MRI

Patellar tendon images were collected immediately following thigh muscle scans. Images were obtained using the Siemens Magnetom Prisma 3T whole-body MRI system combined with a 15-channel Tx/Rx Knee Coil (Siemens). T2-star (T2∗) images were obtained in 4-mm axial slices (no gap) from the distal pole of the patella to the tibial tuberosity, with no interslice gap (TR: 900 ms, TE: 4.3 ms; field of view: 160 × 160, resolution: 320 × 320, voxel size: 0.5 × 0.5 × 4, flip angle: 60°). Tendon CSA was determined by manually circumscribing the patellar tendon at each slice along the tendon length (Horos v3.3.6, www.horosproject.org) [11,12]. Mean signal intensity was also recorded for each slice circumscribed. Region-specific CSAs were determined, as we [11,12] and others [14] have noted that changes in tendon properties are often regional. Tendon CSA was used to calculate stress (force/CSA) to develop stress-strain curves for determining tangent modulus. To minimize the effect of previous physical activity on tendon CSA and signal [34,35], participants were instructed to refrain from exercise programs for ≥48 h before the MRI examinations.

### Statistical analysis

Prior sample size estimations were completed utilizing previous work [[11], [12], [13], [14],^26^] to estimate SD and meaningful differences between study groups. Based on these previous studies, we estimated that a sample size of 12 participants per group would allow us to detect group differences in skeletal muscle volume, quadriceps strength, tendon modulus/stiffness, and mean tendon CSA, with a power of 0.80 or higher and a Cohen’s d of 0.65 or higher (α = 0.05). Participant characteristics were compared using an ordinary 1-way analysis of variance (ANOVA). The remaining data were compared utilizing a 2-way repeated measures ANOVA with training (time) and diet (group) as factors. Multiple comparison testing was used to explore differences further if a significant *P* value was observed in the ANOVA test. We corrected for multiple comparison testing using the Holm-Šídák method. Post hoc, the 2 high protein (1.4 g/kg/d) groups were combined and compared to the normal protein (0.8 g/kg/d) group as a secondary analysis. Significance was set at *P* < 0.05. All results are reported as mean ± SE. We completed the statistical analysis and figure preparation using GraphPad Prism 10 for MacOS version 10.0.0 (GraphPad Software, LLC).

## Results

### Participant characteristics

Before beginning the RT regimen, age and BMI were not different between diet groups (*P* > 0.05, Table 1). We noted modest group differences in body weight and height even though participants were randomly assigned to diet groups (*P* ≤ 0.05, Table 1).

**TABLE 1:** Participant characteristics

	Dietary group		
	Normal protein control 0.8 g·kg^–1^·d^–1^	High protein mixed control 1.4 g·kg^–1^·d^–1^	High protein beef experimental 1.4 g·kg^–1^·d^–1^
Age (y)	65 ± 1	66 ± 2	67 ± 3
Prebody weight (kg)	75.9 ± 4.1	69.4 ± 2.9	82.1 ± 3.7^1^
Height (cm)	167 ± 1	160 ± 2^2^	164 ± 2
BMI (kg/m^2^)	27 ± 1	27 ± 1	31 ± 2
Menu adherence (%)	93 ± 2	92 ± 2	91 ± 3
Adherence to protein-rich foods (%)	96 ± 6	95 ± 5	93±8

Menu adherence reflects the percentage of assigned menu items consumed by a participant. Adherence to protein-rich foods reflects the percentage of assigned protein-rich foods consumed by a participant. Values are presented as mean ± SE.

1 *P* ≤ 0.05, 1.4 g**·**kg^–1^**·**d^–1^ control compared with 1.4 g**·**kg^–1^**·**d^–1^ beef.

2 *P* < 0.05, 0.8 g**·**kg^–1^**·**d^–1^ control compared with 1.4 g**·**kg^–1^**·**d^–1^ control.

### Diet adherence

Based on the dietary recalls, participants’ habitual total daily protein intake at baseline was not different between groups (*P* > 0.05, normal protein control: 0.81 ± 0.11 g protein/kg/d; high protein-control: 0.87 ± 0.06 g protein/kg/d; high-protein beef (0.88 ± 0.14 g protein/kg/d). During the 12-wk intervention period, adherence to consuming the proscribed menu-specific foods and the protein-rich foods were high (91%–93% and 93%–96%, respectively) and not different between groups (*P* > 0.05, Table 1). Actual weekly protein intake was consistent with group assignments (Table 2). Leucine, glycine, and proline intakes were greater in the groups consuming 1.4 g protein/kg/d compared to 0.8 g protein/kg/d control (*P* < 0.05). Further, leucine, glycine, and proline intakes were greater in the 1.4 g protein/kg/d beef group compared to the 1.4 g protein/kg/d control (*P* < 0.05). Consistent with greater protein intake, blood urea nitrogen increased (*P* < 0.05) in the 1.4 g protein/kg/d control (pre: 16 ± 1 mg/dL; post: 20 ± 2 mg/dL) and 1.4 g protein/kg/d beef (pre: 13 ± 1 mg/dL, post: 16 ± 1 mg/dL) groups, but not in the group consuming 0.8 g protein/kg/d (*P* > 0.05, pre: 16 ± 1 mg/dL, post: 15 ± 1 mg/dL).

**TABLE 2:** Subject daily intakes

	Normal protein control 0.8 g·kg^–1^·d^–1^	High protein mixed control 1.4 g·kg^–1^·d^–1^	High protein beef experimental 1.4 g·kg^–1^·d^–1^
Leucine (g/d)	4.7 ± 0.3	7.3 ± 0.3^1^	9.1 ± 0.5^1^^,^^2^
Glycine (g/d)	2.4 ± 0.2	3.7 ± 0.2^1^	4.9 ± 0.3^1^^,^^2^
Proline (g/d)	4.4 ± 0.2	6.0 ± 0.2^1^	7.1 ± 0.3^1^^,^^2^
Daily carbohydrate intake (g/d)	300.7 ± 6.1	254.4 ± 5.0^1^	261.9 ± 4.3^1^
Daily fat intake (g/d)	85.4 ± 2.1	71.8 ± 1.9^1^	76.6 ± 2.1^3^
Daily protein intake (g/d)	63.3 ± 3.3	94.3 ± 4.4^1^	115.0 ± 5.6^1^^,^^2^
Daily protein intake (mg/kg body weight)	0.82 ± 0.01	1.38 ± 0.02^1^	1.41 ± 0.2^1^

Daily intakes of specific nutrients are based on analysis of the rotating weekly diet menus for each group.

Compared with 1.4 g**·**kg^–1^**·**d^–1^ beef, values are presented as mean ± SE.

1 *P* < 0.001, 0.8 g**·**kg^–1^**·**d^–1^ control compared with 1.4 g**·**kg^–1^**·**d^–1^ control and 1.4 g**·**kg^–1^**·**d^–1^ beef.

2 *P* < 0.05, 1.4 g**·**kg^–1^**·**d^–1^ control compared with 1.4 g**·**kg^–1^**·**d^–1^ beef.

3 *P* < 0.05, 0.8 g**·**kg^–1^**·**d^–1^ control.

### Muscle strength and volume

**FIGURE 2:**
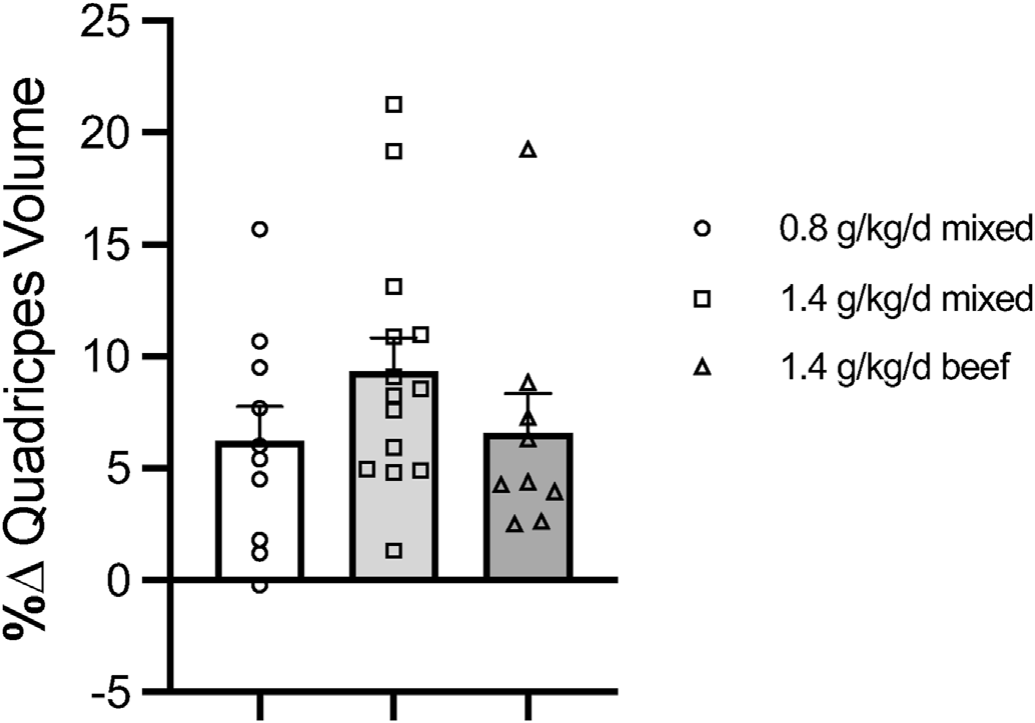
Percent change in quadriceps muscle volume. Data presented as mean ± SE. Circles, squares, and triangles represent individual data points for each subject within a diet group.

Leg extension, leg press, leg curl, chest press, and seated row 1RM increased with training regardless of dietary group (30–42% increase, Table 3, *P* < 0.0001, main effect of training). Leg extension strength normalized to quadriceps volume also increased with training independent of diet (Table 3, *P* < 0.0001, main effect of training). No differences in strength gains were noted between dietary groups (*P* > 0.05, Supplemental Table 1). Quadriceps muscle volume increased with training (*P* < 0.0001, Table 4, main effect). The change in quadriceps volume was not different between dietary groups (*P* > 0.05, Figure 2, Supplemental Table 2). Retrospectively, we combined the 2 high protein groups and completed a second ANOVA analysis comparing total protein intakes, 0.8 compared with 1.4 g/kg/d. No difference in the response to RT between dietary groups was detected after combining the 2 high protein groups (*P* > 0.05).

**TABLE 3:** Skeletal muscle strength assessments

Dietary group	Leg extension (kg)		Leg extension (kg)/quadriceps volume (cm^3^)		Leg press (kg)		Leg curl (kg)		Chest press (kg)		Seated row (kg)	
	Pre	Post^1^	Pre	Post^1^	Pre	Post^1^	Pre	Post^1^	Pre	Post^1^	Pre	Post^1^
Normal protein control 0.8 g·kg^–1^·d^–1^	92 ± 5	130 ± 4	0.14 ± 0.1	0.19 ± 0.0	176 ± 12	232 ± 17	68 ± 4	96 ± 5	61 ± 4	85 ± 6	98 ± 6	129 ± 5
High protein mixed control 1.4 g·kg–1·d–1	92 ± 5	130 ± 6	0.18 ± 0.0	0.23 ± 0.0	167 ± 10	246 ± 15	62 ± 4	81 ± 5	63 ± 6	83 ± 6	94 ± 4	122 ± 5
High protein beef Experimental 1.4 g·kg^–1^·d^–1^	103 ± 11	136 ± 10	0.17 ± 0.0	0.23 ± 0.0	196 ± 18	244 ± 14	70 ± 7	88 ± 5	63 ± 4	82 ± 4	107 ± 6	134 ± 5
Main effect of training	96 ± 4	132 ± 2	0.17 ± 0.0	0.22 ± 0.0	180 ± 8	241 ± 4	67 ± 2	88 ± 4	63 ± 1	83 ± 1	100 ± 4	128 ± 4

Values are presented as mean ± SE. Sample sizes with both pre- and postmeasurement were 11 for the 0.8 g**·**kg^–1^**·**d^–1^ control group, 14 for the 1.4 g**·**kg^–1^**·**d^–1^ control group, and 9 for the 1.4 g**·**kg^–1^**·**d^-1^ beef group.

1 *P* < 0.001, main effect of training.

**TABLE 4:** Quadriceps and patellar tendon cross-sectional area assessments

Dietary group	Quadriceps muscle volume (cm^3^)		Mean patellar tendon CSA (mm^2^)		Body weight normalized CSA (mm^2^/kg^0.667^)		Proximal tendon CSA (mm^2^)		Midtendon CSA (mm^2^)		Distal tendon CSA (mm^2^)	
	Pre	Post^1^	Pre	Post	Pre	Post	Pre	Post^1^	Pre	Post^1^	Pre	Post
Normal protein control 0.8 g·kg^–1^·d^–1^	645 ± 29	680 ± 30	121 ± 5	121 ± 5	6.8 ± 0.3	6.8 ± 0.3	129 ± 6	135 ± 7	122 ± 4	121 ± 7	117 ± 6	110 ± 4^2^
High protein mixed control 1.4 g·kg^–1^·d^–1^	534 ± 30	582 ± 31	108 ± 3	111 ± 3	6.4 ± 0.2	6.6 ± 0.2	119 ± 5	125 ± 4	104 ± 3	109 ± 3	102 ± 3	105 ± 4
High protein beef Experimental 1.4 g·kg^–1^·d^–1^	586 ± 52	625 ± 56	110 ± 2	114 ± 2	5.8 ± 0.2	6.1 ± 0.1	119 ± 3	123 ± 3	107 ± 3	116 ± 3	106 ± 3	106 ± 4
Main effect of training means	589 ± 32	629 ± 28	113 ± 4	115 ± 3	6.4 ± 0.3	6.5 ± 0.2	123 ± 4	128 ± 4	111 ± 6	115 ± 4	108 ± 4	107 ± 2

Sample sizes with both pre- and postmeasurement: 11 for the 0.8 g·kg^-1^·d^–1^ control, 14 for the 1.4 g·kg^–1^·d^–1^ control, and 9 for the 1.4 g·kg^–1^·d^–1^ beef; tendon: 10 for the 0.8 g·kg^–1^·d^–1^ control, 14 for the 1.4 g·kg^–1^·d^–1^ control, and 8 for the 1.4 g·kg^–1^·d^–1^ beef.

Abbreviation: CSA, cross-sectional area.

1 *P* < 0.05, main effect of training, values presented as mean ± SE.

2 *P* < 0.05, decrease with training, values presented as mean ± SE.

### Patellar tendon CSA and signal

**FIGURE 3:**
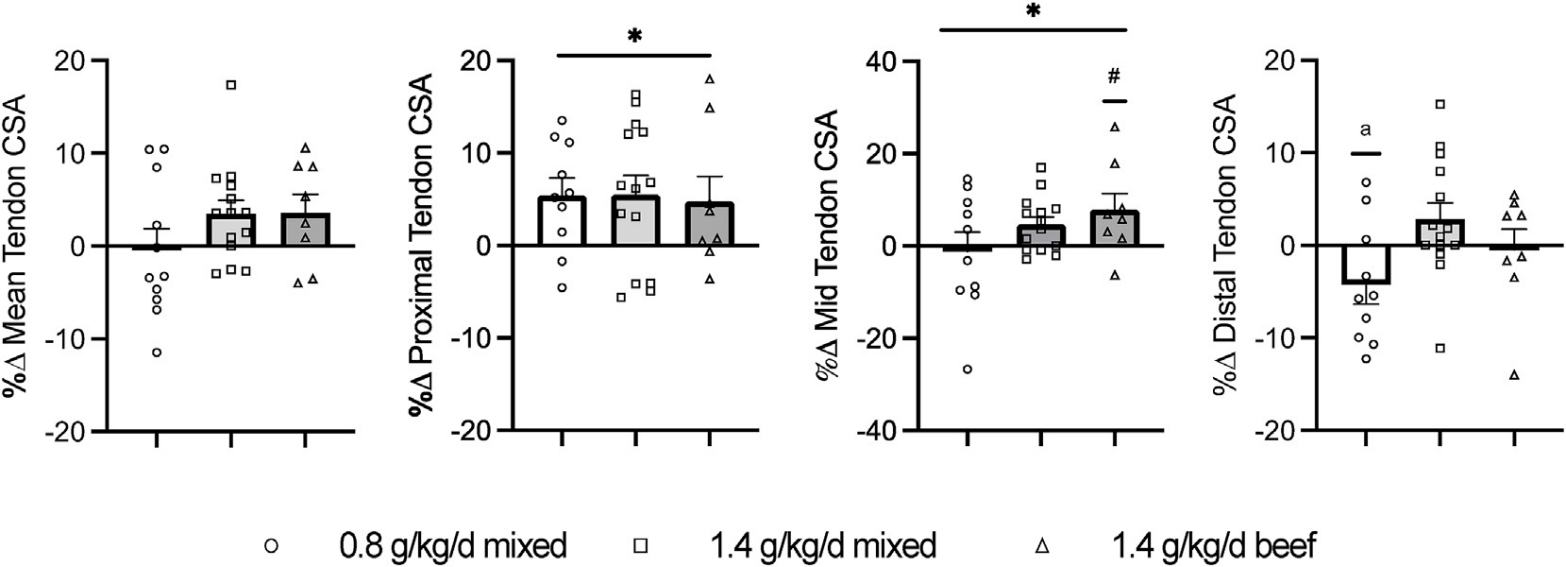
Percent change in mean and regional patellar tendon cross-sectional area (CSA). Data presented as mean ± SE. Circles, squares, and triangles represent individual data points for each subject within a diet group. ∗*P* ≤ 0.05, the main effect of time. ^#^*P* < 0.10, increase in the 1.4 g/kg/d group. ^a^*P* < 0.05, a decrease in the 0.8 g/kg/d group.

We noted a trend for a training effect on mean tendon CSA (*P* = 0.07, main effect, Table 4), but the change with training was not different between dietary groups (*P* > 0.05, Figure 3, Supplemental Table 3). Neither training nor diet affected body weight normalized CSA (*P* > 0.05, Table 3). In contrast, proximal tendon CSA increased with training (*P* < 0.05, Table 4) with no difference in the response between dietary groups (*P* > 0.05, Table 4, Figure 3). Midtendon CSA increased with training (*P* = 0.05, main effect). The increase in midtendon CSA tended (*P* < 0.1) to be greater in the group consuming 1.4 g protein/kg/d with beef than 0.8 g protein/kg/d (Figure 3). No difference between the 2 higher protein groups was observed in the midtendon CSA change (*P* > 0.05). Lastly, we found a significant interaction effect for distal patellar tendon CSA (*P* < 0.05, Table 4). Post hoc analysis revealed a decrease in distal CSA in the 0.8 g protein/kg/d (*P* < 0.05, Figure 3) but no change with training in the 1.4 g protein/kg/d groups (*P* > 0.05, Figure 3). The patellar tendon T2∗ signal was not different with training in any dietary group (*P* > 0.05, Table 5). Combining the high protein groups did not alter the findings, except for midtendon CSA. In this case, midtendon CSA increased with training only in those consuming 1.4 g/kg/d (*P* < 0.05).

**TABLE 5:** Patellar tendon MRI T2-star signal

Dietary group	Mean patellar tendon T2∗ signal (AU)		Proximal tendon T2∗ signal (AU)		Midtendon T2∗ signal (AU)		Distal tendon T2∗ signal (AU)	
	Pre	Post	Pre	Post	Pre	Post	Pre	Post
Normal protein control 0.8 g·kg^–1^·d^–1^	0.55 ± 0.07	0.48 ± 0.05	0.77 ± 0.13	0.71 ± 0.09	0.47 ± 0.06	0.41 ± 0.03	0.46 ± 0.04	0.40 ± 0.06
High protein mixed control 1.4 g·kg^–1^·d^–1^	0.42 ± 0.03	0.48 ± 0.03	0.55 ± 0.05	0.61 ± 0.03	0.37 ± 0.04	0.44 ± 0.04	0.41 ± 0.04	0.46 ± 0.03
High protein beef Experimental 1.4 g·kg^–1^·d^–1^	0.44 ± 0.05	0.43 ± 0.04	0.62 ± 0.11	0.60 ± 0.07	0.35 ± 0.03	0.37 ± 0.04	0.45 ± 0.03	0.42 ± 0.03

Values are presented as mean ± SE. Sample sizes with both pre- and postmeasurement were 10 for the 0.8 g·kg^–1^·d^–1^ control group, 12 for the 1.4 g·kg^1^·d^–1^ control group, and 8 for the 1.4 g·kg^–1^·d^–1^ beef group.

Abbreviations: AU, arbitrary units; T2∗, T2-star.

### Patellar tendon biomechanical properties

Patellar tendon properties at peak voluntary force are presented in Table 6, Supplemental Table 4. We did not note training or diet effects on peak patellar tendon modulus, strain, or stress (*P* > 0.05). Biomechanical data were also normalized to a common force (Table 7). As with the peak data, neither training nor diet affected patellar tendon biomechanical properties when normalized to a common force (*P* > 0.05).

**TABLE 6:** Patellar tendon biomechanical properties at peak voluntary force

Dietary group	Modulus (GPa)		Strain (%)		Stress (MPa)	
	Pre	Post	Pre	Post	Pre	Post
Normal protein control 0.8 g·kg^–1^·d^–1^	0.77 ± 0.11	0.76 ± 0.12	6.33 ± 0.58	7.31 ± 1.19	16.87 ± 1.32	19.34 ± 2.02
High protein mixed control 1.4 g·kg^–1^·d^–1^	0.64 ± 0.10	0.65 ± 0.06	5.94 ± 0.84	6.42 ± 0.83	15.04 ± 2.03	17.12 ± 2.34
High protein beef Experimental 1.4 g·kg^–1^·d^–1^	0.79 ± 0.05	0.67 ± 0.11	7.93 ± 1.03	9.95 ± 0.07	17.84 ± 3.39	21.04 ± 4.05

Values are presented as mean ± SE. Sample sizes with both pre- and postmeasurements were 9 for the 0.8 g·kg^–1^·d^–1^ control group, 11 for the 1.4 g·kg^–1^·d^–1^ control group, and 5 for the 1.4 g·kg^–1^·d^–1^ beef group.

**TABLE 7:** Patellar tendon biomechanical properties normalized to a common force pre-to-post training

Dietary group	Modulus (GPa)		Strain (%)		Stress (MPa)	
	Pre	Post	Pre	Post	Pre	Post
Normal protein control 0.8 g·kg^–1^·d^–1^	0.73 ± 0.09	0.68 ± 0.09	5.85 ± 0.53	6.70 ± 1.23	14.60 ± 1.30	14.95 ± 1.51
High protein mixed control 1.4 g·kg^–1^·d^–1^	0.56 ± 0.08	0.55 ± 0.07	5.23 ± 0.60	5.56 ± 0.73	12.53 ± 1.73	12.61 ± 1.75
High protein beef Experimental 1.4 g·kg^–1^·d^–1^	0.72 ± 0.12	0.55 ± 0.10	6.60 ± 0.84	8.36 ± 1.92	14.88 ± 2.40	14.81 ± 2.40

Values are presented as mean ± SE. Sample sizes with both pre- and postmeasurement were 9 for the 0.8 g·kg^–1^·d^–1^ control group, 11 for the 1.4 g–kg^–1^·d^–1^ control group, and 5 for the 1.4 g·kg^–1^·d^–1^ beef group.

## Discussion

The current study addressed whether greater daily protein intake, emphasizing lean beef, would enhance tendon and skeletal muscle adaptations to a 12-wk RT program compared to normal and high protein control diets. Primary skeletal muscle outcomes were quadriceps muscle volume and 1RM strength. Primary patellar tendon outcomes included tendon stiffness/modulus and MRI-determined patellar tendon CSA and T2∗ signal. Inconsistent with our hypotheses, we did not note a differential effect of a higher protein diet emphasizing beef on our primary outcomes.

Progressive declines in skeletal muscle and tendon health are a part of the aging process of humans [36], resulting in reduced quality of life. Older women are particularly vulnerable to sarcopenia; thus, we focused our current research on this population. To our knowledge, this is the first investigation to evaluate the effect of greater protein intake on concurrent skeletal muscle and tendon adaptations to chronic RT, focusing solely on older women. Other strengths of our study include careful dietary control, high menu adherence, supervised RT sessions, and combined tendon and skeletal muscle assessments.

### Muscle mass and strength

Recent meta-analyses suggest greater protein intake is associated with improved physical function [37], greater lean body mass, and increased muscle size and strength [17,[38], [39], [40]]. Nunes et al. [38] found that a greater habitual protein intake (∼1.6 g/kg/d), similar to our work’s protein intake, is associated with modest improvements in lean body mass and muscle strength when combined with RT. However, whether such gains can be realized in older adults has been debated [41]. Interestingly, an increase in total daily protein consumption from mixed sources or predominately from beef did not enhance RT adaptations in the skeletal muscles of older adult women compared to 0.8 g/kg/d, even with the greater leucine intake noted in the 1.4 g protein/kg/d beef group.

Anabolic signaling in response to amino acids is impaired in older adults compared to young individuals [19], which could account for the reduced effect of a higher protein diet on skeletal muscle outcomes. A reduced anabolic response to protein is consistent with a recent systematic review by Morton et al. [42]. The authors [42] concluded that although protein supplementation improved RT-induced changes in muscle strength and size, the effect of protein intake declined with increasing age. Further, Unterberger et al. [39] noted no difference in physical function, handgrip strength, and muscle quality outcomes after increasing daily protein intake to 1.5–1.6 g/kg body weight/d during an RT intervention for older adults.

### Patellar tendon properties

The health of tendons has not been considered in the context of protein intake and aging. As with skeletal muscle, tendons change with aging, including increased nonenzymatic cross-linking [10]. Changes in tendon CSA and biomechanical properties have been reported but with inconsistent results and differences between tendons (e.g., Achilles compared with patellar) [[10], [11], [12],[43], [44], [45]]. Regardless, RT [14] or regular sport-specific loading [46] increases patellar tendon stiffness and CSA in younger adults, but such adaptations may be limited in older adults [12]. Preclinical studies in rodents [25] and a single study in humans [26] support the use of protein nutrition to promote tendon adaptations. Therefore, we assessed the impact of greater habitual protein intake on tendon morphology and biomechanical properties.

#### Patellar tendon morphology

We have previously reported a lower T1 MRI signal in older adults compared to young adults [11]. However, we did not observe an effect of training or diet on patellar tendon MRI T2∗ signal in the current investigation. RT can alter tendon structure; thus, it is possible that the single T_E_ imaging approach was not sensitive enough to detect modest changes with exercise or diet. Newer MRI methodologies, including multi-T_E_ enhanced T2∗, correlate well with age-related [47] and structural [33,48] changes in tendons and should be utilized in future studies.

We noted a trend for an increase in patellar tendon mean CSA with RT, but differences between dietary groups did not reach statistical significance. However, when evaluating region-specific CSA, we found that RT increased proximal patellar tendon CSA (∼5%) in all groups. A main effect of RT on midtendon CSA was noted, primarily limited to the high protein group, which consumed more beef. Surprisingly, distal tendon CSA decreased in the 0.8 g/kg/d group, with no change noted in the higher protein groups. A similar increase in proximal patellar tendon CSA was reported in young adults completing a similar 12-wk RT regimen [14]. The authors also found an increase in distal but no change in midtendon CSA [14]. More recently, Eriksen et al. [49] reported that a 12-mo RT program with older adults resulted in tendon hypertrophy, primarily in the proximal and mid portions of the patellar tendon.

These limited studies suggest that greater loading during an RT intervention may be required to realize an increase in tendon CSA, particularly in older adults. In our previous work [12], in which no change in CSA was observed, 3 sets of 10 repetitions (70% of 1RM) of leg extension exercises were completed 3 times a week for 12 wk. In contrast, Kongsgaard et al. [14] and Eriksen et al. [49] employed a protocol of 4 sets each of leg extension and press for 4 sets of 10 repetitions (70% of 1RM). Although the lower workload in our previous work was sufficient to induce skeletal muscle hypertrophy, a larger overall training load may be needed to observe concomitant changes in patellar tendon CSA.

Although there was no strong effect of greater daily protein intake, our findings suggest that greater protein consumption may be a strategy to enhance tendon hypertrophy with RT. Regional measures of tendon CSA tended to favor higher protein intake. Our analysis of the weekly diets (Table 2) shows that leucine and glycine intake was greater in the 1.4 g/kg/d protein groups than in the 0.8 g/kg/d group and was further elevated when beef was a main source of dietary protein. In young adults, increased circulating glycine and proline were associated with increased collagen synthesis [50] and increased leucine intake, which has been shown to have an anabolic effect on the patellar tendon [26]. Farup et al. [26] provided young adults with a high-leucine whey protein hydrolysate during a 12-wk RT program. Whey protein resulted in a greater increase in skeletal muscle and proximal patellar tendon CSA. In contrast to our feeding regime, Farup et al. [26] provided their protein intervention on training days, with half provided before and half after each training session.

As discussed above, in the context of skeletal muscle, timing of protein ingestion may also be necessary for promoting tendon adaptations. In future work, combining different nutritional interventions to maximize tendon hypertrophy should also be considered. Collagen peptide supplementation during 14 wk of RT resulted in greater increases in Achilles tendon CSA and muscle thickness compared to a placebo [51]. Thus, combining collagen peptide supplementation with greater protein intake may enhance RT-induced tendon hypertrophy.

#### Patellar tendon biomechanical properties

To our knowledge, this is the first investigation to assess tendon biomechanical properties in older women after a combined RT program and dietary protein intervention. As with previous work with older adults [12], RT did not alter patellar tendon modulus, stress, or strain. Further, greater protein intake or beef consumption did not influence our assessment of patellar tendon biomechanical properties. The lack of an increase in tendon modulus suggests that our intervention did not impact the material properties of the patellar tendon and that appropriate changes in the tendon extracellular matrix accompanied the increase in CSA. Although we noted tendon hypertrophy with RT and greater protein intake, these changes did not translate to changes in biomechanical properties. Our results suggest that further work is needed to discover approaches to preserve and improve tendon biomechanical properties as we age. Although life-long exercise may aid in maintaining tendon properties [52], recent work by Eriksen et al. [49] found that even 12 mo of heavy RT in older adults only induced modest changes in patellar tendon CSA and no change in modulus.

## Limitations

Some possible limitations to our study should be considered when interpreting our findings. The modest sample size for tendon biomechanical testing, particularly in the group consuming more beef, may have limited our ability to detect differential responses in these variables. We have previously reported the variability associated with the tendon CSA and mechanical property assessments [11,12], which can average ≤8% for tendon modulus. We acknowledge that the inherent variability in the in vivo assessment and the modest sample size in the groups likely limited our ability to observe significantly different responses in tendon properties between groups.

Our post hoc power analysis, using prestudy assumptions and realized sample sizes, suggests that an increase in sample size would have likely allowed for better differentiation between RT and diet effects, particularly with mean patellar tendon CSA (with loss of power due to sample attrition as high as 20%). Surprisingly, we did not find a significant effect of greater daily dietary protein intake on most muscle and tendon parameters. Additionally, the large number of statistical tests performed are subject to an inflated family-wise error rate, such that those findings that were indicated as statistically significant have a higher type I error rate than the individual test cut-off at 5%. However, given the small sample sizes compounded by some sample attrition, we present the results as is with the caveat that additional work is needed to support these findings.

It is possible that the timing of protein intake limited the impact of increased protein intake on our skeletal muscle outcomes [53]. A review by Morton et al. [42] found a beneficial effect of greater protein intake on skeletal muscle outcomes. Most of the studies included in the meta-analysis provided some or all of the additional protein shortly after each RT session to maximize the impact of protein intake on postexercise protein synthesis. Many acute studies demonstrate that enhanced postexercise muscle protein synthesis after resistance exercise provides a large protein bolus, usually immediately after each training session. Stimulation of muscle protein synthesis after resistance exercise is correlated with increasing blood amino acid concentrations, particularly leucine [54]. Allocating the greater protein intake throughout the day rather than providing a large postexercise bolus may not have consistently elevated blood amino acids to the degree that optimized our population’s anabolic response to resistance exercise [55]. Instead, we chose an approach consistent with the habitual behavior of older adults, largely dispersing protein intake over 3 meals.

### Future directions

In future work, it would be helpful to determine if protein intake timed immediately after resistance exercise would improve tendon adaptations to chronic training. We have recently demonstrated that oral consumption of amino acids can increase the local peritendinous concentration of amino acids [21]. However, the concentration of individual amino acids in the peritendinous space after bolus administration was lower than those obtained in skeletal muscle after a similar bolus of amino acids. Although we did not measure peritendinous amino acids in the current study, it is likely that the overall exposure of the tendon to amino acids with the increased dietary protein intake was less than in skeletal muscle. If, as in skeletal muscle, increasing amino acids results in higher protein synthesis rates, a further increase in protein intake and subsequent elevation of peritendinous amino acids would result in a greater anabolic response. Future work should also evaluate the impact of varied amino acid concentrations on tendon collagen synthesis and cellular activation within the extracellular matrix. Further, assessing the role of individual amino acids in modulating tendon extracellular matrix would also be useful, given the apparent benefits of glycine in preclinical models [23,24] and leucine in young adults [26].

In conclusion, we hypothesized that a healthy diet high in protein emphasizing lean beef would augment improvements in skeletal muscle and tendon outcomes to RT compared to healthy control diets with normal or high protein and less total red meat. Consistent with previous studies in older adults [13], chronic RT improved muscle strength and volume but did not change patellar tendon biomechanical properties. In contrast to previous work with older adults [13], we noted a significant increase in proximal patellar tendon CSA with RT. Although we observed improvements in skeletal muscle and regional tendon CSA with training (main effects), greater daily protein intake predominantly from unprocessed lean beef did not differentially affect our primary outcomes, including skeletal muscle volume and strength. Further, greater daily protein consumption, predominately from lean beef, had no significant effect on the patellar tendon outcomes compared to our control diets.

## Author contributions

The authors’ responsibilities were as follows – CCC and WWC: designed research; CCC, NWCC, RLL, SEP, CMG, HMW, ACB, JMV, LSS, CJG, and CR: conducted research; CCC, NWCC, SEP, CMG, HMW, ACB, MAF, and ZJH: analyzed data or performed statistical analysis; CCC: wrote the article; CCC: had primary responsibility for final content; and all authors: read and approved the final manuscript.

## Funding

This study was funded in part by the National Cattlemen’s Beef Association, a contractor to the Beef Checkoff, to CCC and WWC; Purdue University Office of the Executive Vice President for Research and Partnerships COVID-19 Disruption Funds to CCC. The NIH grant S10 OD012336 partly supported the MRI. Menu development was supported by the Indiana Clinical and Translational Sciences Institute, funded in part by Award Number UM1TR004402 from the NIH, National Center for Advancing Translational Sciences, and Clinical and Translational Sciences Award. CCC and NWCC received salary support from the USDA National Institute of Food and Agriculture, Hatch project 7000704.

## Data availability

Data for results presented in this manuscript will be made available upon request.

## Conflict of interest

During the time this research was conducted, WWC received funding for research from NIH, USDA, Beef Checkoff, Foundation for Meat and Poultry Research and Education, Pork Checkoff, North Dakota Beef Commission, Barilla Group, Mushroom Council, National Chicken Council, and the Whey Protein Research Consortium. All other authors report no conflicts of interest.
