# Supplementary material for: Greater Protein Intake Emphasizing Lean Beef Does Not Affect Resistance Training-Induced Adaptations in Skeletal Muscle and Tendon of Older Women: A Randomized Controlled Feeding Trial
Source: J Nutr. 2024 Apr 9;154(6):1803–14. doi: 10.1016/j.tjnut.2024.04.001 (PMC11923425; doi:10.1016/j.tjnut.2024.04.001)
Supplement: multimedia component 1 [file mmc1.docx]

**Supplemental Table 1: Percent Change in One-Repetition Maximum**

| **Dietary Group** | **Leg Extension (%Δ)** | **Leg Extension (kg)/Quadriceps Volume (%Δ)** | **Leg Press (%Δ)** | **Leg Curl (%Δ)** | **Chest Press (%Δ)** | **Seated Row**  **(%Δ)** |
| --- | --- | --- | --- | --- | --- | --- |
| Normal Protein Control  0.8 g·kg^-1^·day^-1^ | 46±8 | 38±8 | 35±9 | 42±6 | 39±6 | 33±4 |
| High Protein Mixed Control  1.4 g·kg^-1^·day^-1^ | 45±9 | 33±8 | 45±6 | 32±6 | 38±9 | 32±4 |
| High Protein Beef Experimental  1.4 g·kg^-1^·day^-1^ | 38±11 | 29±11 | 30±9 | 33±11 | 33±6 | 27±4 |

Values are presented as mean ± standard error.

**Supplemental Table 2: Percent Change in Quadriceps Volume and Patellar Tendon CSA**

| **Dietary Group** | **Quadriceps Muscle Volume (%Δ)** | **Average Patellar Tendon CSA (%Δ)** | **Proximal Tendon CSA (%Δ)** | **Mid Tendon CSA (%Δ)** | **Distal Tendon CSA (%Δ)** |
| --- | --- | --- | --- | --- | --- |
| Normal Protein Control  0.8 g·kg^-1^·day^-1^ | 5.6±1.5 | -0.4±2.2 | 5.4±1.9 | -1.1±4.1 | -4.3±2.1 |
| High Protein Mixed Control  1.4 g·kg^-1^·day^-1^ | 9.3±1.5 | 3.5±1.4 | 5.5±2.1 | 4.7±1.6 | 2.9±1.8* |
| High Protein Beef Experimental  1.4 g·kg^-1^·day^-1^ | 6.6±1.7 | 3.6±2.0 | 4.8±2.7 | 7.9±3.5 | -0.5±2.2 |

*p<0.05. 0.8 g•kg^-1^•day^-1^ mixed protein vs. 1.4 g•kg^-1^•day^-1^ mixed protein. Values presented as mean ± standard error. Cross-sectional area (CSA)

**Supplemental Table 3: Percent Change in Patellar Tendon Magnetic Resonance Imaging T2* Signal**

| **Dietary Group** | **Average Patellar Tendon T2* Signal (%Δ)** | **Proximal Tendon T2* Signal (%Δ)** | **Mid Tendon T2* Signal (%Δ)** | **Distal Tendon T2* Signal (%Δ)** |
| --- | --- | --- | --- | --- |
| Normal Protein Control  0.8 g·kg^-1^·day^-1^ | -8.3±7.2 | -0.4±9.9 | -11.4±6.9 | -11.8±8.8 |
| High Protein Mixed Control  1.4 g·kg^-1^·day^-1^ | 17.7±6.2 | 18.8±9.5 | 22.0±6.7 | 16.0±6.1 |
| High Protein Beef Experimental  1.4 g·kg^-1^·day^-1^ | 3.1±7.9 | 12.7±14.4 | 13.8±10.3 | -6.2±2.8 |

Values presented as mean ± standard error. Arbitrary Units (AU).

**Supplemental Table 4: Percent Change in Patellar Tendon Biomechanical Properties**

| **Dietary Group** | **Peak Modulus (%Δ)** | **Peak Strain (%Δ)** | **Peak Stress (%Δ)** | **Force Normalized Modulus (%Δ)** | **Force Normalized Strain (%Δ)** |
| --- | --- | --- | --- | --- | --- |
| 0.8 g•kg^-1^•day^-1^  mixed protein | 2.0±11.7 | 15.0±14.0 | 14.9±9.3 | -4.7±10.2 | 14.7±15.8 |
| 1.4 g•kg^-1^•day^-1^  mixed protein | 23.4±11.9 | 16.5±14.8 | 26.7±14.8 | 7.4±12.0 | 15.0±16.0 |
| 1.4 g•kg^-1^•day^-1^  beef protein | 3.3±29.5 | 19.3±19.4 | 34.7±41.5 | -21.3±9.6 | 25.8±21.8 |

Values presented as mean ± standard error.
